# Lessons Learned From the Stakeholder Engagement in Research: Application of Spatial Analytical Tools in One Health Problems

**DOI:** 10.3389/fvets.2020.00254

**Published:** 2020-05-13

**Authors:** Kaushi S. T. Kanankege, Nicholas B. D. Phelps, Heidi M. Vesterinen, Kaylee M. Errecaborde, Julio Alvarez, Jeffrey B. Bender, Scott J. Wells, Andres M. Perez

**Affiliations:** ^1^Department of Veterinary Population Medicine, College of Veterinary Medicine, University of Minnesota, St. Paul, MN, United States; ^2^Department of Fisheries, Wildlife and Conservation Biology, College of Food, Agriculture and Natural Resource Sciences, University of Minnesota, Minneapolis, MN, United States; ^3^Minnesota Aquatic Invasive Species Research Center, University of Minnesota, Minneapolis, MN, United States; ^4^Centro de Vigilancia Sanitaria Veterinaria (VISAVET), Universidad Complutense, Madrid, Spain; ^5^Departamento de Sanidad Animal, Facultad de Veterinaria, Universidad Complutense, Madrid, Spain; ^6^Environmental Health Sciences, School of Public Health, University of Minnesota, Minneapolis, MN, United States

**Keywords:** transdisciplinary research, case studies, co-creation of knowledge, risk maps, risk communication, epidemiology, one health, veterinary research

## Abstract

Stakeholder engagement in research is widely advocated as a tool to integrate diverse knowledge and perspectives in the management of health threats while addressing potential conflicts of interest. Although guidelines for stakeholder engagement exist in public health and environmental sciences, the feasibility of actionable decisions based on scientific analyses and the lessons learned from the stakeholder engagement in the process co-creation of knowledge have been rarely discussed in One Health literature and veterinary sciences. Risk maps and risk regionalization using spatiotemporal epidemiological/analytical tools are known to improve risk perception and communication. Risk maps are useful when informing policy and management decisions on quarantine, vaccination, and surveillance intended to prevent or control threats to human, animal, or environmental health interface (i.e., One Health). We hypothesized that researcher-stakeholder engagement throughout the research process could enhance the utility of risk maps; while identifying opportunities to improve data collection, analysis, interpretation, and, ultimately, implementation of scientific/evidence-based management and policy measures. Three case studies were conducted to test this process of co-creation of scientific knowledge, using spatiotemporal epidemiological approaches, all related to One Health problems affecting Minnesota. Our interpretation of the opportunities, challenges, and lessons learned from the process are summarized from both researcher and stakeholder perspectives. By sharing our experience we intend to provide an understanding of the expectations, realizations, and “good practices” we learned through this slow-moving iterative process of co-creation of knowledge. We hope this contribution benefits the planning of future transdisciplinary research related to risk map-based management of One Health problems.

## One Health and Spatial Epidemiology

One Health encompasses the collaborative multidisciplinary approach of improving health and well-being through prevention of risks and mitigation of the effects of crises that originated at the interface of ecosystems, animals, and humans (1–3). A commonality among One Health problems is the need to understand patterns of spread of health threats over space and time (4, 5), which can be addressed using common scientific approaches such as spatial epidemiology (6, 7). Spatiotemporal epidemiological/analytical tools are useful in identifying those patterns of spread and quantifying the association of the patterns with underlying risk factors (8–10). Integration of epidemiological concepts, data, statistics, spatial analysis, and geographic information system (GIS) enables achieving these objectives (11–13).

## Estimation of Risks Using Spatial Epidemiological Tools

Risk is defined as the probability that an event with negative consequences occurs, as well as the magnitude of those negative consequences (14). The application of spatiotemporal analytical tools on existing data enables hypothesizing and predicting the spread of adverse events in relation to underlying factors (8, 15, 16). These analyses often results in risk maps, which are useful risk communication tools as discussed in diverse fields including health, disaster management, and econometrics (17). Risk maps are two- or three-dimensional visualizations depicting high-risk (i.e., disease hot spots) or risk-free areas, and may include the third dimension of time depending on the data and analyses used (11, 13). This process of risk regionalization has multiple advantages when designing interventions such as surveillance programs or management strategies to prevent and control harmful agents (18–20).

## Stakeholder Engagement and Risk Communication Using Maps

Stakeholders are “any person or group who has an interest in the Research Topic and/or who stands to gain or lose from a possible policy change that, directly or indirectly, might be influenced by the research findings” (21). The stakeholder engagement is “an iterative process of actively soliciting the knowledge, experience, judgment, and values of individuals selected to represent a broad range of direct interest in a particular issue, for the dual purposes of: creating a shared understanding; making relevant, transparent and effective decisions” (22). Here onwards, we use the term “research/er” to denote spatiotemporal modeling outputs and the university-based scientists, whereas, the organizations or personnel who would potentially use resulting risk maps for decision/policy-making purposes will be referred to as “stakeholders.”

While frameworks and guidelines for stakeholder engagement and co-creation of knowledge exist in public health and environmental sciences (22–28); the feasibility of actionable decisions based on scientific analyses, and the lessons learned from the process has been rarely documented in One Health literature and veterinary sciences. This lack of documentation is attributable to the iterative nature of researcher-stakeholder interactions, involvement of multiple stakeholders, and the complexities of financial and socio-political aspects related to the decision and policy-making process (29–31). The stakeholder engagement process can ensure that researchers are contextualizing the decision-making environment and understanding the complex nature of decision-making, policy, and program implementation for a given issue (32–34). Hence, co-creation of knowledge in community-based research collaborations, i.e., collaborative knowledge generation by researchers working alongside stakeholders, is described to have broader societal impact (33). In essence, the stakeholder engagement allows for setting decision-oriented analytical goals, creating actionable knowledge together (33, 35), and prompting practical questions—“are these findings applicable” and “so what”?

We hypothesized that researcher-stakeholder engagement throughout the research process could enhance the utility of risk maps in scientific/evidence-based management and policy measures in Minnesota while enhancing the data quality. In this paper, we discuss three case studies in which One Health problems in Minnesota were addressed using spatiotemporal epidemiological/analytical tools jointly with relevant local stakeholders in order to develop risk maps. A summary of the studies found in Table 1. By sharing our experience and perspectives, our objective is to provide an understanding of the “good practices” of this slow-moving iterative process of co-creation of evidence-based knowledge.

**Table 1:** Comparison of the three case studies.

**Case study/ Specific aim**	**Ecosystem health: modeling the spatial dynamics of invasive zebra mussels and Eurasian watermilfoil in Minnesota**	**Animal health: epidemiological characterization of Johne's disease in Minnesota dairy cattle**	**Animal and human health: spatiotemporal patterns of historic animal Anthrax outbreaks in Minnesota**
Reference for further details	(36–38)	(39)	(38, 40)
Causative agent/s	Zebra mussels (a bivalve) and Eurasian watermilfoil (an aquatic plant)	*Mycobacterium avium* subsp. *Paratuberculosis* (a bacteria)	*Bacillus anthracis* (a bacteria)
Host population/s	Waterbodies	Dairy cattle	Livestock, wildlife, and human
Regulations	Reportable	Non-reportable	Reportable
Data source and the primary stakeholder	Minnesota Department of Natural Resources	Minnesota Dairy Herd Improvement Association	Minnesota Board of Animal Health
Stakeholders primary objectives of data collection	Passive surveillance	Testing and record keeping	Passive surveillance
Stakeholder's objectives in using risk maps	Focused risk-based surveillance for early detection of invasions	Potential defining of risk zones	Inform decisions on area of vaccination zones
Number of meeting participants	11 (University of Minnesota *n* = 5 and Stakeholders *n* = 6)	7 (University of Minnesota *n* = 4 and Stakeholders *n* = 3)	6 (University of Minnesota *n* = 3 and Stakeholders *n* = 3)
**SIMILARITIES AMONG CASE STUDIES**			
Common characteristics	• Causative agent/s are endemic to the state of Minnesota • Cause harm to ecosystem, animal, or human health • Cause substantial economic losses • Require attention to improve mitigation strategies • The adverse effects inflicted and proposed solutions are in alignment with One Health objectives • Have no or minimal existing scientific method to quantify the risk of spread • Have existing databases collecting incidence data through passive surveillance or voluntary testing		

## The Process of Stakeholder Identification and Interaction

In One Health, identification of the stakeholders who will contribute to the decision/policy making activities is challenging, due to both the multi- and transdisciplinary nature of the interactions (29). Here, the target group of stakeholders were defined based on [1] their interest in the research process, [2] their involvement in past research with the University of Minnesota, [3] their commitment to providing data and expertise, and [4] their potential as upper-level stakeholders, such as state government personnel who have the decision-making power for the problems studied here and capable of integrating analytical outputs to inform intervention decisions. Hence, the interactions were transdisciplinary in nature where collaborative knowledge generation by researchers was done in partnership with relevant stakeholders.

The stages of co-creation of knowledge has been extensively discussed in public health (33) and environmental sciences (27). In alignment with the steps described by Djenotin and Meadow (27); our stages of stakeholder engagement involved [1] setting-up, [2] development and design, [3] implementation of research and communication, and [4] output management and dissemination. According to the taxonomy of co-creation of knowledge (25, 33, 41), the stakeholder engagement process here was primarily “Mode 1” (i.e., university-based scientific knowledge was generated) with shared objectives of “Mode 2” where application oriented spatiotemporal analyses were conducted based on stakeholder inputs.

**Figure 1:**
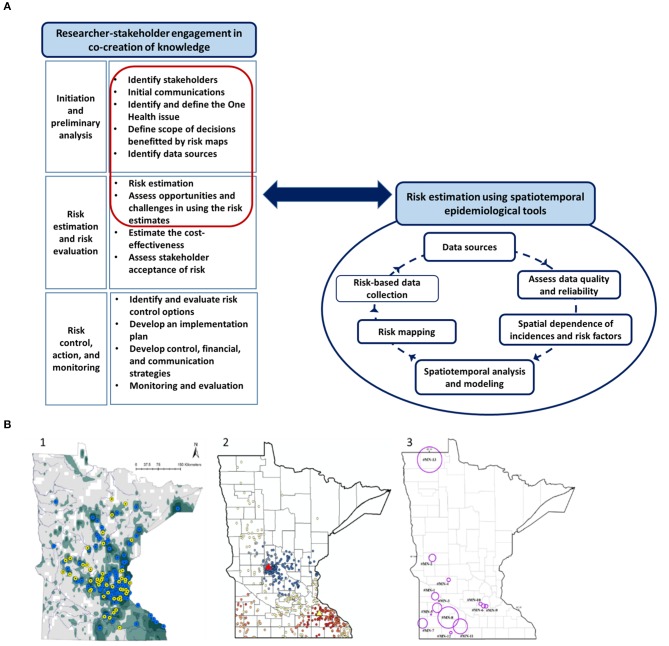
The use of spatial analytical tools in three One Health problems affecting Minnesota. **(A)** The steps in the process of using spatiotemporal analytical tools in risk communication and evidence-based management of One Health problems. The red highlighted steps related to risk estimation and recognizing opportunities and challenges are discussed as “lessons learned” from the researcher-stakeholder engagement in the paper. **(A)** Illustration was generated as part of the current study. **(B)** Maps related to the three case studies presented. (1) Risk map depicting the probability of introduction of invasive Eurasian watermilfoil into Minnesota waterbodies (37); (2) Map depicting the result of Getis Ord Gi* spatial analysis of “disease free status” of Johne's disease (39); and (3) Map illustrating the spatiotemporal clusters of animal Anthrax in Minnesota during the 1912 through 2014 (40). Please refer to the original publications for further details on the spatial analytical techniques and higher resolution of the maps.

Spatiotemporal analysis and researcher-stakeholder meetings of all three case studies were conducted by the lead author with the support from coauthors. Therefore, the objectives of the researcher-stakeholder meetings, the mannerism of conduct, note-taking, feedback process, and the interpretations were comparable across the studies. Each study involved frequent interactions and consultation with relevant stakeholders since the setting-up stage. Studies were conducted from 2014 through 2018. The initial communications with stakeholders facilitated identification research goals. To this end, in-person meetings and email communications were used to gather data, understand the existing management process, and the potential for a risk-map based approach to help design, implement and optimize the interventions. It is important to note that the discussions presented as “lessons learned” were mainly based on the final in-person meetings with stakeholders. Our research team met with the relevant stakeholders (3–6 stakeholders, depending on the case; Table 1) for pre-planned final meetings that lasted for 3 to 4 h. The meeting agenda was shared a week prior to the meetings and informed consent was obtained from all participants prior to the meetings to share the discussion content anonymously. Notes were taken at each meeting and summary reports/meeting minutes were shared among participants for transparency and clarity allowing them to add any missing details. Each meeting was initiated by researchers with a presentation that summarized study objectives, data, spatiotemporal analytical methods, assumptions, output risk maps, epidemiologically important risk factors, and strengths and limitations (Figure 1). Then the meetings were opened for discussions under four key topics as follows:

1. Opportunities for the use of risk maps to inform decision-making.
2. Opportunities for spatiotemporal analysis to improve data collection and analysis.
3. Challenges in using evidence-based risk maps from the stakeholder's perspective.
4. Challenges in the research communication process from the researcher's perspective.

## The Case Studies

### Ecosystem Health: Determining High-Risk Areas for Aquatic Invasions

Zebra mussels (*Dreissena polymorpha*) and Eurasian watermilfoil (*Myriophyllum spicatum*) are aggressive aquatic invasive species (AIS) that harm water resources and require costly preventive measures (42). These AIS are reportable to the Minnesota Department of Natural Resources (MNDNR), which is the state government agency responsible for the management of AIS. Spatial analytical tools were used to understand the spread of AIS, reporting patterns, and to develop maps depicting high-risk areas for invasions (36–38). This risk regionalization may inform resource allocation for early detection, surveillance, and watercraft inspection (37). Staff from the University of Minnesota Aquatic Invasive Species Research Center, state government personnel including MNDNR officials, county representatives, and watershed district managers participated in the final discussion.

### Animal Health: Epidemiological Characterization of Johne's Disease in Minnesota Dairy Cattle

Johne's disease (JD), caused by *Mycobacterium avium* subsp. *paratuberculosis* (MAP), is a chronic animal disease (43). While oft-debated, potential for foodborne transmission of MAP offers one justification for tracking JD (44), another is to help dairy producer decision-making in reducing the economic loss due to JD, such as when purchasing cattle (45). Surveillance of JD in the U.S. is challenging due to lack of regulatory requirements, imperfect diagnostics, and associated expenses including time and labor (46, 47). Yet, some dairy producers participate in voluntary JD testing programs. We explored the use of a voluntary JD testing program implemented by the Minnesota Dairy Herd Improvement Association (MNDHIA) as a passive surveillance tool to measure JD status in Minnesota (39). Spatial analytical tools were used to understand the representativeness of the data, spatial spread of JD, and association of the disease status with underlying risk factors (39). The interventions that may benefit from the regionalization included defining “test-negative areas,” i.e., risk-zoning of JD, was seen as a first step in introducing a flexible and producer-driven JD testing program. JD is not subjected to regulatory requirements and MNDHIA as a testing and record-keeping agency has direct interaction with dairy producers who could benefit from research findings. Therefore, the analytical results were discussed with the members of the MNDHIA instead of the Minnesota Board of Animal Health (MNBOAH) (i.e., the state governmental agency for animal health).

### Animal and Human Health: Spatiotemporal Patterns of Historic Animal Anthrax Outbreaks in Minnesota

Anthrax, caused by bacterium *Bacillus anthracis*, is a reportable zoonotic disease affecting animals and humans (48). MNBOAH leads the data collection and informs farmers regarding animal vaccination and human health concerns. Spatiotemporal analytical tools were used to detect patterns of Anthrax progression, intensity, direction, and recurrence (disease hot spots) (40). The interventions that may benefit from the spatial analysis included defining surveillance/vaccine radii for animal Anthrax (40). The study suggested that past outbreaks spread within a range of radii between 2 and 40 km (40). The findings were regarded as informative and supportive of the existing, expert opinion based (49), guidelines on Anthrax prevention. The UMN research team and members of MNBOAH participated in the discussions.

## The Benefits of Combining Stakeholder Engagement and Spatiotemporal Analysis: Opportunities Summarized

While each study was unique, the co-creation of knowledge based on stakeholder engagement facilitated identification and narrowing the research goals to be addressed using spatial analysis, identifying the problems related to data, and guiding the choice of analytical tools accordingly. The resulting risk maps informed the discussions among meeting participants and provided a rationale to implement map-based targeted surveillance and interventions.

The stakeholder engagement guided the researchers' choice of spatial analytical tools, which in turn resulted in suggestions for improvement and validation of the existing databases. For example, based on the stakeholder's input on passive surveillance of AIS, the spatial pattern recognition conducted on AIS was directed to identify reporting biases and the analysis indicated that AIS reporting was highly correlated with human population density (36). Hence, researchers chose spatial analytical tools that could account for reporting biases in the next steps when developing risk maps for AIS (37). For the study on JD, because there was no existing analysis on the distribution and determinants of JD, both researchers and stakeholders agreed that it was critical to assess the distribution of MNDHIA participants (*n* = 600) and how representative they were of Minnesota dairy herds (*n* = 4,746) (39). Similarly, for the Anthrax study, researchers modified the spatiotemporal scan window sizes and conducted a sensitivity analysis to justify the used parameters based on the stakeholder engagement and their experience on the extent of past Anthrax outbreaks (40). Both the JD and Anthrax study results were analyzed in relation to the underlying environmental risk factors. A key database improvement discussed related to both JD and Anthrax studies was the importance of geocoding the exact location where animals were housed instead of using their primary farm location, for more accurate analyses.

Capacity building within stakeholder groups and enabling them to understand the use of spatiotemporal analytical tools was an added benefit of this process. While spatial analysis offered solutions to the One Health problems at the population level, issues like JD were intrinsically associated with individual animal level management of the disease (45). Therefore, the stakeholder engagement process further supported identifying the importance of analyzing the system as a whole by combining other epidemiological tools including population modeling and cost-effective analysis with spatial analyses when addressing One Health problems.

## Challenges: Stakeholder's Perspective

Challenges related to AIS included the mismatch of expectations in predicting real-time invasion risks. While the models provided an accurate “snapshot” estimate of risk, the dynamic nature of the system demanded the importance of temporal component to prioritize risk. Time was not incorporated into the analysis attributable to the quality of data (37). To provide alternative risk thresholds, maps were divided into five consecutive categories [Figure 1B; (37)]; however, it was challenging to agree upon a cut-off risk level/threshold, because at each risk rank there was a trade-off of “sensitivity” (correctly determining high-risk areas) and “specificity” (correctly determining low-risk areas). This subjective nature of the acceptable levels of sensitivity and specificity was seen as a limiting factor that may lead to the restricted use of spatiotemporal analytical tools when informing decisions. The importance of measuring “how good” the spatial analytical tools were in predicting invasions by means of model validation techniques and measures of predictive powers was emphasized (8). The challenges were also discussed in the light of the absence of a measure of success to “convince” the public and emphasizing the importance of the suggested control activities and inability to answer the question of “who takes the responsibility?” for any changes in the resource allocation. By narrowing down the research goals, the risk estimates were available only for two major AIS, hence, stakeholders identified the challenge in proposing to redistribute resources based on two AIS while several more are affecting Minnesota waters.

Challenges related to the JD study included the absence of estimates of cost-effectiveness if the dairy producers were encouraged to implement JD testing based on the map based test-positive or test-negative areas. The difficulty to motivate producers for JD testing due to the lack of regulatory requirements and inability to measure the success of control measures as a tool for “convincing” the producers was discussed. Another challenge identified was the privacy concerns when displaying JD status by the farm on maps, especially if the maps were to be available for producers/public via an online platform. Researchers offered the option for smoothing out the geographical representation of risk areas instead of mapping each farm as a solution.

In the Anthrax study, identifying the parameters of space-time windows used in the spatiotemporal cluster analysis (50) was a discussion topic between stakeholders and researchers. This impacts the detection of outbreaks of varying cluster sizes and affects vaccination response efforts (40). Researcher's adjusted the scan window sizes and conducted a sensitivity analysis responding to practical perceptions from the stakeholders. While Anthrax is reportable and vaccination is recommended, promoting vaccination was a hurdle due to lack of indemnity and the absence of recent outbreaks, which made disseminating knowledge on Anthrax control a lesser of a priority.

Side-by-side comparisons of cost-benefit of the suggested risk map-based modifications compared to the status quo was emphasized during all the meetings. A cost-benefit analysis was important for these upper-level stakeholders to communicate with their respective funding agencies and the beneficiaries of the decisions when proposing to use evidence-based approaches over the traditional criteria of resource allocation (34).

## Challenges: Researchers' Perspective

Despite regular engagement between stakeholders and researchers, the direct impacts of the spatial tools on policy and interventions were limited. This was partially attributable to the lack of clarity on roles, responsibilities, and rules of engagement of researchers in the co-creation process to influence the decisions as discussed by Mauser et al. (25). A key challenge researchers faced was communicating the strengths of spatiotemporal analyses while acknowledging the assumptions and limitations of the data and analytical methods (11, 17, 51). This has been seen commonly where the uncertainty of information credited for transparency is interpreted by audiences as incompetence (17, 52).

In JD study, stakeholders emphasized the importance of presenting analytical results reflecting clear, concise, and actionable recommendations that aid producers' action plans. For example, stating “Farms on loamy or clay soil may consider increasing the sand content around the cattle premises to reduce the environmental survival of JD pathogen,” was preferred over presenting the same information as “compared to farms on sandy soil, farms on loam or clay soils were more likely to have JD-positive cows” (39). Research communication steps were relatively simpler in the Anthrax study, given the suggested range for vaccine or intervention radii captured the current recommendations (40).

Across the three studies, a common challenge was setting clear goals and problem statements. While the stakeholder-engagement supported the process of narrowing down and recognizing goals that are feasible to address using spatiotemporal analysis, the translation of these outputs back into real-world scenarios was challenging due to multiple factors. For example, the Minnesota legislature allocates 10-million USD yearly to provide resources for county-based AIS prevention activities, such as education, surveys, and watercraft inspections (53). However, the resources are distributed proportionally to the share of boat ramps and trailer parking spaces in each county (53). Our recommendation to redistribute financial resources in an evidence-based manner was not considered due to the absence of cost-effectiveness estimates and focused on only two AIS.

Although the knowledge was co-created, stakeholder's tendency to make changes in decisions about interventions was slow-moving. For example the suggestion to conduct map-based JD testing faced multiple challenges including the lack of legislative requirements for testing and the absence of communication portals to disseminate maps of disease status in a timely manner. Therefore, while the study identified future steps of research, the immediate influence on informing decisions was slow. Similarly, a conservative approach was taken to maintain the existing recommendations given the analysis of Anthrax suggested a range of distances for surveillance/vaccination radii that included the current ring vaccination radius (49).

## Lessons Learned and Suggestions for “Good Practices”

Three case studies were conducted to understand the use of spatiotemporal analytical tools as a unifying scientific approach that is applicable in One Health problems. To improve the impact of the analyses, relevant stakeholders were engaged throughout this process of co-creating evidence-based knowledge. The key lessons learned were: [1] stakeholder-engagement and co-creation of knowledge is a slow-moving and an iterative process that requires both parties to understand the achievable goals and have realistic expectations (34); [2] early stakeholder engagement supported setting clear expectations, shared goals and choosing suitable spatiotemporal analytical tools; [3] it is important to communicate scientific outputs in a simple manner that support decision-making; [4] existing data provides valuable first steps in data-driven risk assessment, while recognizing opportunities to improve data quality; [5] analyzing the cost-effectiveness of proposed changes compared to status quo is essential to informing the decision-making process, and [6] clarifying roles, responsibilities, and rules of engagement of both researchers and stakeholders is essential when co-creating knowledge (25).

As described in the literature of public health and environmental sciences (25, 27, 33), co-creation of knowledge through early engagement of stakeholders in transdisciplinary One Health research facilitated identification of problems that could be addressed using spatial epidemiology. While risk maps improved risk communication and stimulated conversations to inform decision-making (17), as discussed in multiple health and environmental studies (25, 27, 30, 54), our experience also emphasized the importance of training both researchers and stakeholders in science communication for a sustainable researcher-stakeholder relationship. As discussed by Manlove et al. (55), One Health and veterinary sciences are likely to cite within their own disciplines. Thus, this study may contribute to bridging between existing literature and terminology on co-creation of knowledge with One Health and Veterinary sciences.

While this perspective paper contributes to the literature of co-creating knowledge in One Health and veterinary research, the limitations of our approach include: engaging only with selected primary stakeholders, limited comparisons our approach with parallel topics such as participatory epidemiology (56) and responsible research and innovation (57), and not conducting follow-up conversations with stakeholders. While the primary stakeholders chosen here may provide a leadership role in the decision-making process, admittedly, when addressing One Health problems, it is ideal to bring together all the relevant multisectoral stakeholders (28, 58). This step would provide a more inclusive approach which ensure researchers considered all view points, while supporting the decision making process for all affected stakeholders.

Despite the process of co-creation of knowledge, stakeholders' tendency to change decisions on health interventions using risk maps is a slow-moving and iterative process attributable to financial and regulatory constraints. This is because “Planning, execution, dissemination, and implementation of research are not separate and linear phases but interwoven.” (33). As suggested by Oliver and Cairney (34), it is also important that researchers understand the do-and-don't in the process, especially in the absence of evidence to support effectiveness of novel approaches (25). However, as suggested in the literature on health services (59), the proactive linkage would gradually strengthen the trust between researchers and stakeholders and support working together toward evidence-based management of One Health problems in the future. Therefore, as university-based researchers, we encourage the co-creation of knowledge through stakeholder-engagement from the setting-up stage, utility and improvement of existing data, evaluating applications to decision-making, and identifying future research needs.

## Conclusions

In conclusion, the three case studies provide valuable insights on expectations, realizations, and “good practices” in transdisciplinary research of combining spatiotemporal analyses and stakeholder engagement to co-create scientifically-based knowledge. While evidence-based approaches in informing decisions and policy can be a slow-moving process, this adaptive approach of co-creating knowledge is more likely to ensure that research outputs are fit for purpose, acceptable, and valuable for the relevant stakeholders to improve the health of human, animal, and the environment.

## Author Contributions

KK planned and conducted the spatial analyses and was involved in the knowledge translation process and discussions with the stakeholders. NP guided the study on Aquatic invasive species study and the stakeholder discussions with Minnesota Department of Natural Resources. HV and KE edited and reviewed the manuscript and provided inputs related to One Health, co-creation of knowledge, and stakeholder interactions and decision-making process. JA provided expert advice in the spatial analysis of both Johne's disease and Anthrax, edited the manuscript, and participated in the knowledge translation meetings with the relevant stakeholders at Minnesota Dairy Herd Improvement Association and Minnesota Board of Animal Health. JB provided expert guidance in the epidemiological investigation of Anthrax study, edited the manuscript, and participated in the knowledge translation meeting with Minnesota Board of Animal Health. SW provided expert guidance in the epidemiological investigation of Johne's disease study, edited the manuscript, and participated in the knowledge translation meeting with Minnesota Dairy Herd Improvement Association. AP provided expert guidance with spatial analysis in all the three studies, provided feedback, and edited the manuscript.

## Conflict of Interest

The authors declare that the research was conducted in the absence of any commercial or financial relationships that could be construed as a potential conflict of interest.
